# A Collagen Hydrolysate Containing Tripeptides Ameliorates Sarcopenia in Middle-Aged Mice

**DOI:** 10.3390/molecules27092718

**Published:** 2022-04-22

**Authors:** Ji-Eun Kim, Eun-Young Kwon, Youngji Han

**Affiliations:** 1 Department of Food Science and Nutrition, Kyungpook National University, 1370 San-Kyuk Dong Puk-Ku, Daegu 41566, Korea; ths01035@naver.com (J.-E.K.); eykwon@knu.ac.kr (E.-Y.K.); 2 Center for Food and Nutritional Genomics Research, Kyungpook National University, 1370 San-Kyuk Dong Puk-Ku, Daegu 41566, Korea; 3 Center for Beautiful Aging, Kyungpook National University, 1370 San-Kyuk Dong Puk-Ku, Daegu 41566, Korea

**Keywords:** collagen hydrolysate, collagen tripeptide, aging, sarcopenia, muscle loss

## Abstract

Collagen peptide (CP) and collagen tripeptide (CTP) are supplementary health foods that exhibit several biological effects. However, the effects of collagen on age-associated sarcopenia and its underlying mechanisms are unclear. C57BL/6J mice (*n* = 24, 12 months old) were divided into three dietary groups and administered AIN93G (aging control, AC; JA BIO, Suwon, Korea), AIN93G plus 0.2% CP, and AING93G plus 0.2% CTP supplement for 12 weeks. The results indicated that the CP and CTP supplements significantly increased the weight of the quadriceps tibialis anterior and gastrocnemius muscles and reduced body fat. A morphological analysis revealed that the spaces within the muscle cells were tight with attenuated fibrosis following CP and CTP supplementation. Immunohistochemistry was applied and a Western blot analysis was performed to determine the underlying mechanisms. The CTP supplement increased the expression of IGF-1, PI3K/AKT, and mTOR, whereas the CP supplement increased the expression of IGF-1 and AMPK in the gastrocnemius of aging mice. CP and CTP ameliorate age-associated sarcopenia through different mechanisms.

## 1. Introduction

Aging is associated with metabolic, physiological, and functional impairments resulting from age-related changes in body composition. Sarcopenia, a type of muscle loss associated with aging and/or immobility, is characterized by the degenerative loss of skeletal muscle mass, quality, and strength [1]. It is closely associated with immobility, falls, osteoporosis, and fractures [2,3,4]. Moreover, the decrease in muscle mass and function results in reduced physical activity, a decrease in total energy consumption, and weight gain/obesity [5]. This may ultimately lead to metabolic diseases, including decreased lung and cardiovascular function [6,7]. Aging induces changes in the synthesis and sensitivity of the growth hormone, cortisol, and sex hormone [8,9]. These hormones have anabolic effects on muscle protein metabolism. The decline in insulin-like growth factor 1 (IGF-1) and growth hormone levels that occurs in the elderly is strongly associated with an increase in visceral fat, a decrease in muscle mass, and a drop in bone density [10,11]. Moreover, proinflammatory cytokines (tumor necrosis factor alpha (TNF-α), interleukin 1 (IL-1), and interleukin 6 (IL-6)) are likely to be involved in age-related muscle loss in humans, and they promote the degradation of protein and suppress protein synthesis, which results in muscle loss [12].

Collagen is a major protein in animals and is present in the extracellular matrix of skin, bone, tendon, and the vasculature [13]. Collagen forms a triple helical structure that comprises repeating glycine (Gly)-X-Y sequences with various amino acids in the X and Y position [14]. The X and Y position predominantly contains proline (Pro) and hydroxyproline (Hyp) respectively, an amino acid specific to collagen. Many studies have indicated that collagen affects changes in body composition skin-care management and improves bone and muscle strength [15,16,17]. Collagen is digested easily and randomly in the digestive system; however, it is rarely cleaved into bioactive peptides, which are hydrolysates of a protein source [18,19]. Then the enzymatic hydrolysate of collagen was developed to increase the physiological effects on the body. Collagen peptides, bioactive peptides including collagen tripeptide (CTP; tripeptide derived from collagen), have conserved the collagen-specific sequence, Gly-Pro-Hyp (GPH), for collagen hydrolysis and have increased the bioavailability of bioactive peptides [18,20]. Collagen hydrate has been known to be effective in antioxidant processes, skin protection, bone and joint health, wound healing, and so on in animal and human [21,22,23,24,25,26,27]. Thus, collagen hydrolysates have been developed over the past two decades as supplements or cosmeceutical products for use worldwide in the current health food market. Several studies have revealed that collagen improves age-related muscle loss [28,29]; however, the results of using collagen peptides supplement and its underlying molecular mechanism are insufficient. Therefore, we examined the effects of low-molecular-weight collagen peptide (500 Da) compared with high-molecular-weight collagen peptide (3000 Da) on the aging-muscle wasting mechanism in C57BL/6J mice.

## 2. Results

### 2.1. Effects of Collagen on BWG, FER, and Organ Weight

There were no significant differences in body weight or food intake in the collagen groups compared with the aging groups (Table 1). Food and energy intake were significantly decreased in the YC group compared with the AC group; however, there were no significant differences in the values among the aging groups. Compared with organ weight, liver weight was higher in the AC group than in other groups, whereas kidney weight was significantly lower (Table 1).

### 2.2. Effects of Collagen on Muscle Loss in Middle-Aged Mice

**Figure 1:**
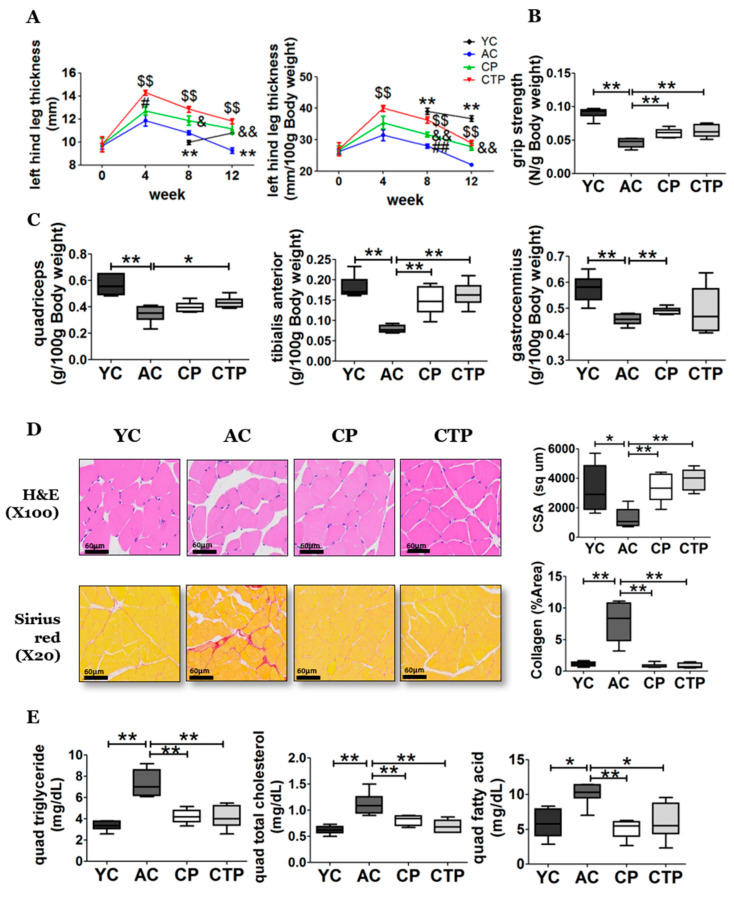
Effect of a 12-week supplementation of CP and CTP on the left-hind-leg thickness, grip strength, muscle weight, and gastrocnemius histopathology. (**A**) Left-hind-leg thickness. The quadriceps (quad), gastrocnemius, and tibialis anterior were sampled. Data are the mean ± SE. Significant differences between YC versus AC are indicated as follows: * *p* < 0.05, ** *p* < 0.01. Significant differences between CP versus AC are indicated as follows: ^&^ *p* < 0.05, and ^&&^ *p* < 0.01. Significant differences between CTP versus AC are indicated as follows: ^$$^ *p* < 0.01. Significant differences between CP versus CTP are indicated as follows: ^#^ *p* < 0.05, ^##^ *p* < 0.01. (**B**) Grip strength (N/g body weight). (**C**) Muscle tissue weight (g/100 g body weight). (**D**) H&E and Sirius red stained transverse-sections of gastrocnemius muscle and fiber cross-sectional area. Scale bar = 60 µm. (**E**) Quadriceps triglyceride, quadriceps total cholesterol, and fatty acid. Data are presented as the mean ± SE. Significant differences are indicated as follows: * *p* < 0.05, and ** *p* < 0.01. YC, young mice control (12 weeks old, *n* = 6); AC, aging control (60 weeks old, *n* = 6); CP, collagen peptide (60 weeks old, *n* = 6); CTP, collagen tripeptide (60 weeks old, *n* = 6).

There was an increase in the thickness of the left hind leg in all aging groups by the fourth week of the experiment. Thereafter, the aging groups experienced an overall loss in thickness of the left hind leg, whereas the YC group experienced an increase by the twelfth week compared with the eighth week. However, among the aging groups, the loss of these values was ameliorated by collagen intake from the fourth week. The thickness of the left hind leg increased significantly from the fourth week in the CTP group compared with the AC group, as well as those of the CP group compared with the AC group from the eighth week (Figure 1A). The whole-body grip strength of the AC group decreased because of aging compared with the YC group, but the reduction of whole-body grip strength of the AC group significantly improved in the collagen-intake group (Figure 1B). As shown in Figure 1C, the weight of the quadriceps, tibialis anterior, and gastrocnemius was significantly decreased in the AC group compared with the YC group. However, the weight of the tibialis anterior and right quadricep muscles in the CTP group was significantly higher compared with that of the AC group. In addition, the weight of the tibialis anterior and gastrocnemius muscles was significantly higher in the CP group compared with that of the AC group. We examined a cross-section of the gastrocnemius muscle fibers to determine whether the muscle mass reduction was a result of a reduction in actual muscle fiber area (Figure 1D). We performed H&E and Sirius red staining of the gastrocnemius to assess the quality of the muscle tissue (Figure 1D). H&E staining revealed that muscle fibers in the AC group were smaller compared with those in the YC group. Furthermore, we observed a tendency for hypertrophic changes and a reduced collagen-occupied region that was increased in the collagen groups (Figure 1D). These results indicate that muscle triglyceride (TG), TC, and FFA levels were significantly increased in the AC group compared with the YC group after 12 weeks of feeding (Figure 1E). This increase in the AC group was suppressed by collagen intake.

### 2.3. Effects of Collagen on Protein Expression Related to Protein Synthesis and Degradation

**Figure 2:**
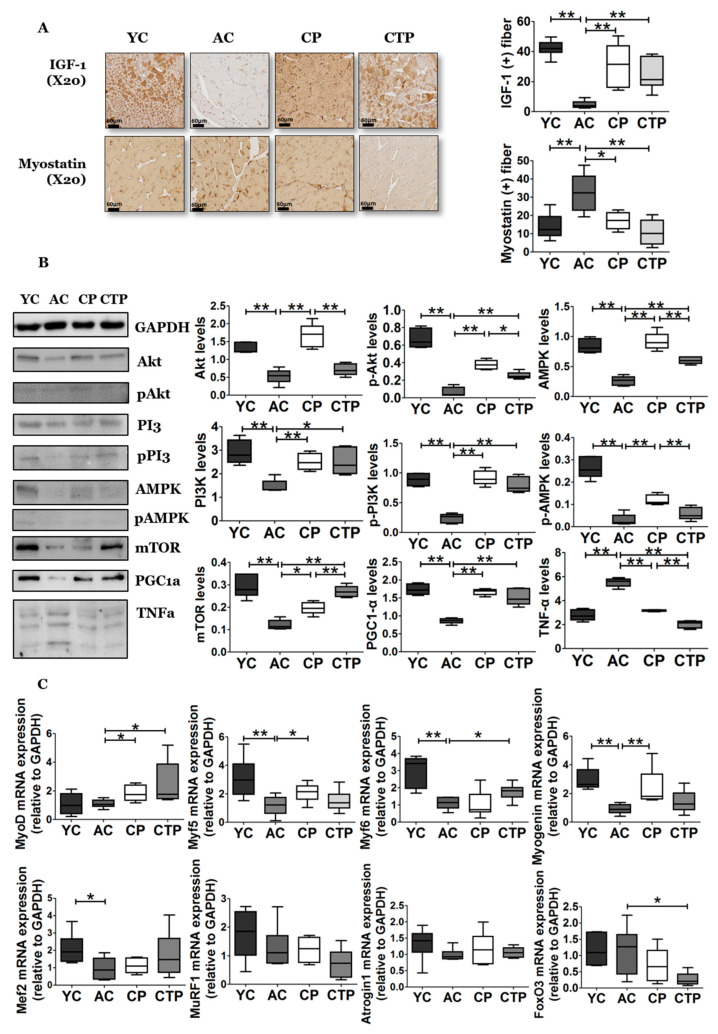
Effect of a 12-week supplementation of CP and CTP on factors related to protein metabolism in the gastrocnemius muscle. (**A**) Immunostaining of the gastrocnemius for insulin-like growth factor 1 (IGF-1) and myostatin. Scale bar = 60 µm. (**B**) Western blot analysis; (**C**) mRNA expression by qRT-PCR. Data are presented as the mean ± SE. Significant differences are indicated as follows: * *p* < 0.05, and ** *p* < 0.01. YC, young mice control (12 weeks old, *n* = 6); AC, aging control (60 weeks old, *n* = 6); CP, collagen peptide (60 weeks old, *n* = 6); CTP, collagen tripeptide (60 weeks old, *n* = 6); MyoD, myoblast determination protein 1; Myf, myogenic factor; Mef, Mouse Embryonic Fibroblasts; MuRF1, muscle RING-finger protein-1; FoxO3, forkhead box O3.

From the immunohistochemical analysis of the gastrocnemius, the expression level of IGF-1 (the upstream factor of protein synthesis) in the gastrocnemius muscle fibers was significantly reduced in the AC group compared with the YC group, whereas a decrease was suppressed by collagen intake (Figure 2A). Moreover, the expression and activation of downstream factors of IGF-1, including PI3K, AKT, and mTOR, were changed in the collagen groups compared with the AC group (Figure 2B), in particular, activated Akt expression in the CTP group compared with the CP group. Furthermore, CPT markedly increased mTOR expression compared with the aging groups. The expression of myostatin (the upstream factor of protein degradation) in gastrocnemius muscle fibers was significantly increased in the AC group compared with the YC group, whereas this increase was suppressed by collagen intake. Specifically, a more significant decrease in myostatin expression was induced in the CTP group compared with the CP group (Figure 2A). The expression analysis (Figure 2C) revealed that the CP supplement significantly increased myoblast determination protein 1 (MyoD), myogenic factor 5 (Myf5), and Myogenin expression compared with the AC group. In the CTP group, MyoD and Myf6 expression were significantly increased, and forkhead box O3 (FoxO3) expression was markedly decreased.

### 2.4. Efficacy of Collagen Adipose Tissue Weight and Muscle Lipid Profile

**Figure 3:**
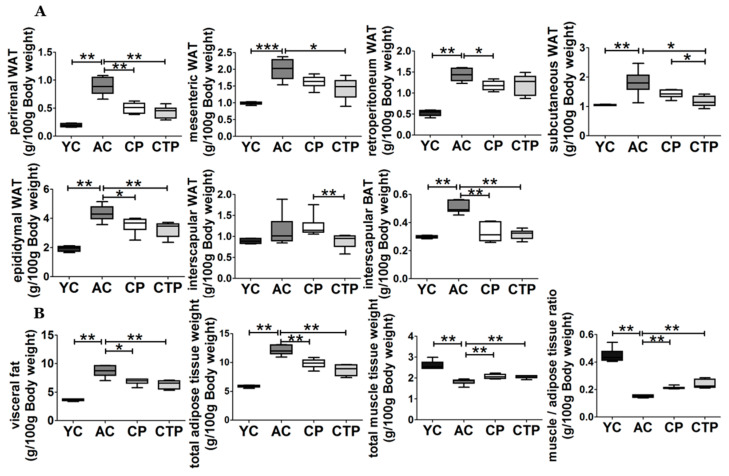
Effect of a 12-week supplementation of CTP and CP on adipose tissue weight (g/100 g body weight), muscle/adipose tissue ratio (g/100 g body weight), and muscle lipid levels. (**A**) adipose tissue weight (g/100 g body weight); (**B**) visceral fat (sum of perirenal WAT, mesenteric WAT, retroperitoneum, and epididymal WAT), total adipose tissue (sum of visceral, subcutaneous and interscapular WAT, and interscapular BAT), total muscle tissue weight (sum of quadriceps, gastrocnemius, tibialis anterior), and muscle/adipose tissue ratio (g/100 g body weight). Data are displayed as the mean ± SE. Significant differences are indicated as follows: * *p* < 0.05, ** *p* < 0.01, and *** *p* < 0.001; YC, young mice control (12 weeks old, *n* = 6); AC, aging control (60 weeks old, *n* = 6); CP, collagen peptide (60 weeks old, *n* = 6); CTP, collagen tripeptide (60 weeks old, *n* = 6).

Figure 3A shows the weight of the adipose tissue among the diet groups. All types of adipose tissue (except intervascular WAT) were significantly increased in the AC group compared with the YC group (Figure 3A,B). This age-induced increase in fat mass in the AC group was suppressed by collagen supplements. In particular, the CTP group exhibited a more significant decrease in mesenteric, retroperitoneal, subcutaneous, interscapular, and epididymal WATs compared with the CP group.

The ratio of total muscle and total adipose tissue weight was calculated to determine the effect of this increase in adipose tissue weight on the muscles. The total muscle tissue weight was not significantly different between the collagen groups and the AC group, whereas that of the AC group was decreased compared with that of the YC group. Similarly, the muscle/adipose tissue weight ratio was significantly lower in the AC group compared with that in the YC group, and this reduction was suppressed by collagen supplementation. In particular, we confirmed that a more significant increase in the ratio was induced in the CTP group compared with the CP group (Figure 3B).

### 2.5. Effects of Collagen on Inflammatory Cytokines, Antioxidant Defense Systems, and G6P Activity

**Figure 4:**
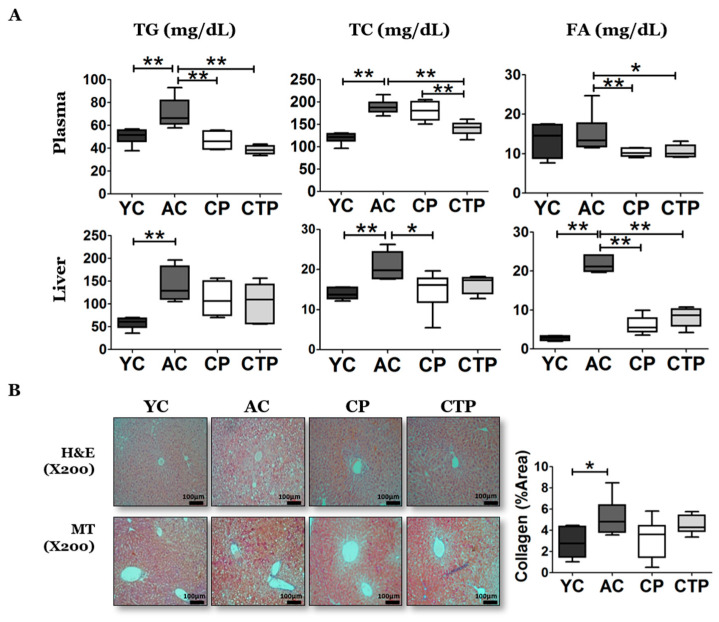
Effect of a 12-week supplementation of CP and CTP on antioxidant metabolism and G6P enzyme activity. (**A**) Cytokine levels. TNF-α, tumor necrosis factor alpha; IL, interleukin. (**B**) Catalase (CAT), glutathione (GSH), GR, glutathione peroxidase (GSH-Px), superoxide dismutase (SOD), and paraoxonase (PON) activities in plasma, erythrocytes, and hepatic tissueData are mean ± SE. Significant differences are indicated as follows: * *p* < 0.05, and ** *p* < 0.01. YC, young mice control (12 weeks old, *n* = 6); AC, aging control (60 weeks old, *n* = 6); CP, collagen peptide (60 weeks old, *n* = 6); CTP, collagen tripeptide (60 weeks old, *n* = 6).

Plasma inflammatory cytokine levels are shown in Figure 4A. The levels of TNF-α, IL-4, and IL-1β were not significantly different between the YC and AC groups; however, the levels of TNF-α and IL-4 were significantly decreased in the CP group compared with the AC group (Figure 4A).

We measured the antioxidant enzyme activities to determine the effects of collagen supplementation. The levels of plasma GSH, PON, hepatic cytosol GSH-Px, GR, liver SOD, and PON were significantly decreased in the AC group compared with the YC group. However, collagen supplementation significantly increased these values (Figure 4B). Erythrocytic CAT and liver GSH levels were similar in the AC and YC groups; however, they were significantly increased by collagen supplementation. Moreover, the hepatic cytosol GSH-Px and plasma PON levels were significantly higher in the CP group compared with the CTP group. In contrast, the levels of plasma PON were significantly higher in the CP group compared with those in the CTP group.

H_2_O_2_ (one of the reactive oxygen species) was significantly increased in red blood cells (RBC) and hepatic cytosol in AC; however, collagen supplementation resulted in a significant decrease. In addition, TBARS levels (an indicator of lipid peroxide content) in RBCs and hepatic mitochondria increased significantly in the AC group compared with the YC group; however, collagen supplementation significantly reduced these values (Figure 4C). Liver G6P activity was significantly decreased in the AC group compared with the YC group; however, G6P activity was significantly increased in the collagen groups compared with the AC group (Figure 4D).

### 2.6. Effects of Collagen on the Biochemistry and Histopathology of the Liver

**Figure 5:**
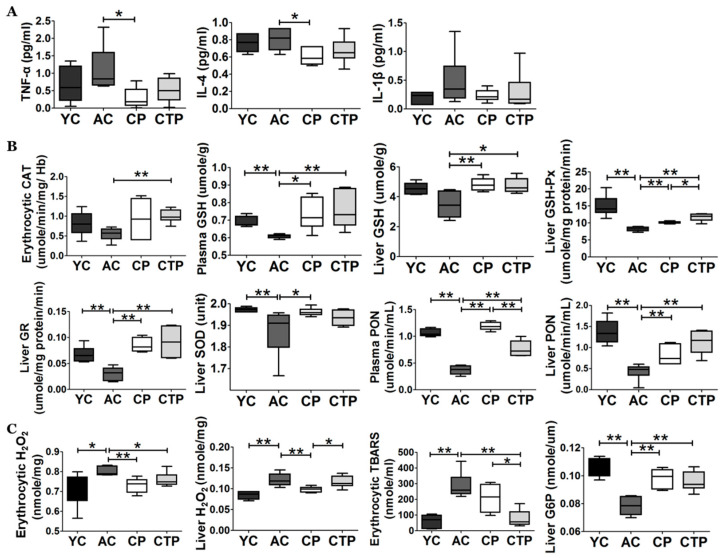
Effect of a 12-week supplementation of CP and CTP on plasma and liver lipid profiles, hepatic tissue morphology, and cytokine levels: (**A**) plasma lipid profiles and liver lipid profiles; (**B**) H&E and MT-stained transverse section of the liver; (**C**) H_2_O_2_ and thiobarbituric acid reactive substance (TBARS) activities in erythrocytes and hepatic tissue and glucose 6 phosphatase (G6P) enzyme activity. Scale bar = 60 µm. Data are the mean ± SE. Significant differences are indicated as follows: * *p* < 0.05, ** *p* < 0.01; YC, young mice control (12 weeks old, *n* = 6); AC, aging control (60 weeks old, *n* = 6); CP, collagen peptide (60 weeks old, *n* = 6); CTP, collagen tripeptide (60 weeks old, *n* = 6).

The AC group exhibited dramatically higher plasma TG and TC levels compared with the YC group; however, this increase was significantly reduced by collagen supplementation. Specifically, the AC group showed no significant differences in plasma FA levels compared with the YC group, although FA levels were significantly decreased after collagen supplementation (Figure 5A). A similar tendency was evident in the lipid profiles of the liver. The AC group showed significantly higher levels of TG, TC, and FA in the liver compared with the YC group, and this increase was significantly ameliorated by collagen supplementation for TC and FA. In particular, TC exhibited a significant reduction in the CP group.

Consistent with these results, H&E staining showed an increase in fats in liver in the AC group compared with that in the YC group, and MT staining showed a significant increase in fibrosis in the AC group compared with that in the YC group. The fibrosis tended to decrease after collagen supplementation (Figure 5B).

## 3. Discussion

In this study, we determined the effect of collagen peptides with different molecular weights on the regulation of overall muscle metabolism in middle-aged mice.

Sarcopenia is caused by aging and a decline in protein synthesis, lack of exercise, neuromuscular degeneration, and an increase in the fat-to-muscle ratio.

Autophagy is essential for maintaining cellular homeostasis (including protein synthesis and skeletal muscle degradation) and for the efficient regulation of cellular responses to stress [30,31]. The proteolytic system in normal skeletal muscle is regulated to preserve components of various organelles [32]. With aging, muscle differentiation decreases and muscle strength weakens, and these are associated with various diseases [33,34]. However, aging suppresses autophagy in skeletal muscle, thereby causing degeneration and weakening of muscle fibers through dysfunctional homeostasis [35]. Insulin and IGF-1 regulate mTOR by activating PI3K and Akt signaling. CP and CTP supplements increased IGF-1 values. Moreover, CP and CTP treatment increased protein synthesis through high IGF-1 levels and the expression of downstream factors, PI3K, Akt, and mTOR [36,37]. Phosphorylated Akt suppresses FoxO3 expression, an important regulator of atrogin1 and MuRF-1 expression [38]. Atrogin1 targets MyoD for degradation through the ubiquitin/proteasome-mediated system. Myostatin potently inhibits myogenesis by reducing MyoD levels [39,40]. Atrogin1 and MuRF-1 are expressed in skeletal muscle for proteolysis, and they play a role in mediating myostatin signaling to regulate myogenesis [38]. Although there was no difference in atrogin1 and MuRF-1 expression in the present study, CP and CTP supplements suppressed protein degradation through the decrease in myostatin and FoxO3 expression. Thus, the CP and CTP supplements increased muscle weight and leg thickness. Altogether, CP and CTP supplements improved age-associated sarcopenia with alterations of molecular signaling through enhanced protein synthesis and suppression of proteolytic processes. Interestingly, CP and CPT were regulated in different ways to ameliorate muscle loss caused by aging. CP supplementation activated PI3K and AMPK and increased mTOR expression compared to the AC group. Moreover, CPT supplement activated the PI3K and AKT and increased the mTOR expression. Moreover, the CPT group activated Akt and expression of mTOR compared to the CP group. It may act as a different signal material depending on the molecular weight of the collagen hydrolysate, and a follow-up study should be performed to identify their acts at the molecular level.

Muscle infiltration with adipose tissue is common and is associated with the loss of skeletal muscle strength and physical function over a diverse set of pathologies [41]. Fat infiltration of muscles has been shown to have a negative impact on muscle strength and mobility [42,43]. Excessive adipose tissue infiltration of skeletal muscle increases with age and has adverse metabolic and mobility effects on the elderly [44]. Previous studies revealed that these fat deposits are associated with aging and inactivity, and perhaps exercise may be able to mitigate this increase in intermuscular adipose tissue [45]. In the present study, we showed that supplementation with CP and CTP suppressed lipid accumulation in the quadriceps muscle with low body fat accumulation. Consistent with these results, another clinical study suggested that collagen peptide supplementation significantly decreased the body fat in overweight adults [16]. A morphologic analysis revealed that muscle fibers were tightly packed in the CP and CTP group compared with the AC group, along with body fat reduction. Similarly, CP and CTP supplementation significantly increased grip strength compared with the middle-aged mice group. Specifically, CP increased AMPK levels and expression in the gastrocnemius muscle. High expression of AMPK in muscle increases energy consumption, decreases markers of skeletal muscle fragility, and increases oxidative capacity through mitochondrial biogenesis [46]. CP significantly decreased inflammatory cytokines levels, and this is consistent with previous results. Furthermore, CP and CTP supplements attenuated the plasma and hepatic lipid contents. The findings suggested that CP and CTP supplement suppressed the muscle lipid accumulation without having side effects on abnormal lipid accumulation.

Free radicals are produced in muscle cells and cause oxidative stress, which leads to aging and cancer [47]. It has been suggested that the accumulation of excessive reactive oxygen species (ROS) induces sarcopenia [48]. Oxidative stress, which is induced by excessive ROS production, can increase proteolysis through the increased expression of the ubiquitin–proteasome system and muscle proteases [49]. An antioxidant supplement may maintain redox status and has the potential to inhibit age-related muscle dysfunction. In the present study, collagen inhibited the increase in the inflammatory response and attenuated antioxidant enzyme activity. In particular, CP significantly increased plasma PON activity. In addition, CTP supplementation significantly increased hepatic GSH-Px activity and decreased TBARs and inflammatory cytokine levels. Moreover, elevated antioxidant efficacy in collagen peptides may contribute to decreased hepatic fibrosis. Consistent with our results, Chiara et al. also reported that collagen peptide supplementation increased the antioxidant enzyme activity [50]. Based on these results, we identified different therapeutic targets of the antioxidant function of collagen by molecular weight.

The limitation of our study should be emphasized. Our experiment was designed without a positive control and used a middle-aged mouse model where aging began. The duration of period in YC group was only 4 weeks. Moreover, we directly measured the weight of adipose tissue not using dual energy X-ray absorptiometry (DXA), which could have been used to have a global view of the body composition of the small animals. In addition, we did not perform a dose-dependent functional evaluation of CP and CTP. Accordingly, another trial is underway in which we will confirm the dose-dependent effect and changes of body composition by CP and CTP supplementation, using DXA, which provides whole-body composition levels more accurately with positive control. We will also use mouse models older than 18 months, with clearly advanced aging, and we will use the same experimental period between the young control group and the aging control group.

## 4. Materials and Methods

### 4.1. Experiment Materials

CP and CTP are average-molecular-weight proteins (3000 and 500 Da, respectively) that were derived from catfish skin gelatin and digested with non-pathogenic Bacillus collagenase-type protease (Amicogen Inc., Jinju, Korea). CP did not contain Gly-Pro-Hyp (GPH); however, CTP contained GPH ≥ 3.2% and collagen tripeptide ≥ 30%. Detailed characterization of the test material was reported by Sontakke et al. in 2016 [18].

### 4.2. Animals and Experimental Design

C57BL/6J mice (*n* = 18, male, 48 weeks old) were purchased from JA BIO (Suwon, Korea). Six male C57BL/6J mice (8 weeks old) were purchased at the 7th week of experiment. The animals were housed in a room with controlled temperature (20–23 °C) and light (alternating between bright and dark for 12 h) and fed with pelletized, unrefined commercial feed for a week after arrival. The old mice were randomly divided into three groups and were fed with each experimental diet for 12 weeks, as indicated in Supplementary Materials Table S1. The groups included an aging control group (*n* = 6, negative control, American Institute of Nutrition AIN93G Semisynthetic Diet), a CP group (*n* = 6, CP, 0.2% CP, supplemented in AIN93G), and a CTP group (*n* = 6, CTP, 0.2% CPT, supplemented in AIN93G). Moreover, the young mice were fed with experimental diet for 12 weeks, as indicated in Table 1 (*n* = 6, YC, American Institute of Nutrition AIN93G Semisynthetic Diet). The mice had free access to water during the experiment, their food intake was recorded daily, and their weight was monitored weekly.

### 4.3. Sampling

Mice were sacrificed by using isoflurane (5 mg/kg BW, Baxter, Deerfield, IL, USA) after a 12-h fast. Blood was collected in heparinized tubes from the vena cava, centrifuged at 1000× *g* for 15 min at 4 °C, and stored at −70 °C before plasma profile analysis. The liver and adipose tissues were removed, rinsed in cold saline, patted dry, weighed, and stored at −70 °C.

### 4.4. Whole Limb Grip Strength Test

A digital force gauge (FGJN.FGP Series, JM Instruments Corp., Seoul, Korea) was used to measure and evaluate the total-limb muscle strength in the mice. To determine total-limb strength, an experiment was performed for each mouse, every day, after five days of practice, and the average maximum limb-strength value was calculated by using the average maximum limb strength (N) divided by body weight in grams (g).

### 4.5. Left Hind Leg Thickness

The left-hind-leg thickness was measured every four weeks of the experiment, using a Blutec digital caliper (BD500) (BLUETEC, Seoul, Korea). To minimize individual differences according to body weight, the left-hind-leg thickness was normalized to 100 g of body weight.

### 4.6. Histopathological and Immunohistochemical Analysis

The liver and gastrocnemius muscle tissues were removed from the mice and fixed in 10% formalin buffer solution. Fixed liver tissue was processed routinely for paraffin embedding, and a 4 μm–thick section was prepared and stained with hematoxylin and eosin (H&E), as well as Masson’s trichrome (MT) staining, and observed under an optical microscope (Nikon, Tokyo, Japan), with a magnification of ×200 [51,52]. Similarly, fixed gastrocnemius muscle tissues were processed routinely for paraffin embedding, and a 4 μm–thick section was prepared and stained with H&E and Sirius red and subjected to immunohistochemistry (insulin-like growth factor 1 (IGF-1), Myostatin). Fibrosis was observed in muscles by Sirius red staining. The slides were observed by using a Moti slide scanner with a magnification of ×20. Histopathological and immunohistochemical analyses were performed based on previously published methods [53,54]. Determination of fiber cross-sectional area and the collagen area was converted into black-and-white and black percentage of included area evaluated by using ImageJ measure command [55].

### 4.7. Plasma, Hepatic, and Muscle Lipid Profile Analysis

Enzymatic analysis of plasma triglyceride (TG) and total cholesterol (TC) was performed by using commercial kits (Asan Pharm Co., Seoul, Korea). Free fatty acid (FFA) was also measured by using an enzymatic assay (ABcam, Cambridge, MA, USA). After extracting hepatic and muscle lipids 22, the dried lipid residues were dissolved in 1 mL of ethanol for triglyceride, TC, and FFA measurements. For emulsification, Triton X-100 and sodium cholate solution were added to 200 µL of dissolved lipid solution. Triglyceride, TC, and FFA were measured by using the same kit as that used for plasma lipid analysis.

### 4.8. Cytokine Analysis

The concentrations of inflammatory cytokines, including TNF-α, interleukin 1β (IL-1β), and interleukin 4 (IL-4), were measured in plasma samples. These cytokines were measured with the Merck Milliplex^®^ MAP kit Mouse cytokine/chemokine magnetic bead panel—Immunology Multiplex Assay (MCYTOMAG-70K, Merck, Billerica, MA, USA), following the manufacturer’s instructions. Plasma from each mouse (30 µL) was assayed in duplicate.

### 4.9. Enzyme Activity

#### 4.9.1. Antioxidant Enzyme Activity

Superoxide dismutase (SOD) activity was determined by measuring the autoxidation of pyrogallol in an alkaline state by the method of Marklund et al. (1974), but with some modifications. Catalase (CAT) activity was measured by a modified method of Abei et al. (1969). Paraoxonase (PON) activity was measured by using a modified method of Mackness et al. (1991). Glutathione reductase (GR) activity was measured by the method of Pinto and Bartley (1969), by measuring the oxidation of NADPH. Finally, total GSH content was determined by using the Ellman method [56].

#### 4.9.2. Lipid Peroxide Content

The erythrocytic lipid peroxide content was measured by using the method of Tarladgis et al. (1964). The amount of 2-thiobarbituric acid reaction substance was calculated by the malondialdehyde extinction coefficient. Cytosolic and mitochondrial hydrogen peroxide (H_2_O_2_) levels in the liver and erythrocytes were measured by Wolff’s method.

#### 4.9.3. G6P Analysis

The hepatic microsomal fraction was used to measure glucose-6-phosphorylase (G6P). G6P activity was determined by using the method of Alegre et al. [57].

### 4.10. Real Time-PCR

Total RNA was isolated and reverse-transcribed into cDNA (complementary DNA). For real-time PCR analysis, template cDNA was diluted in RNAse-free water and used at a concentration of 25 ng/µL. A QuantitTeck SYBR Green PCR kit (QIAGEN, Hilden, Germany) was used for the measurement of mRNA. Gene-specific primers for measuring the expression of each mRNA were synthesized by Genotech Co., Ltd. (Daejeon, Korea). The composition of the reaction included 10 µL of SYBR Green, 2 µL of template, 200 µM of each primer, and RNAse free water to a final volume of 20 µL. The reaction program was as follows: 15 s at 94 °C, and 30 s at 58 and 72 °C. The pre-denaturation step was 2 min at 94 °C. Then 35 subsequent cycles were performed for 30 s at 94 °C, 30 s at 52 or 56 °C, and 30 s at 72 °C, followed by a final extension step for 7 min at 72 °C. The threshold cycles (Ct) were analyzed for each reaction, and the mRNA expression between each experimental group was determined by the CFX96 Real-Time System (Bio-Rad, Richmond, CA, USA). GAPDH was used as an internal housekeeping gene, and the following genes were analyzed: Atrogin1, FoxO3, Mef2, Myf5, Myf6, MyoD, Myogenin, and MuRF1. The primer sequences are listed in Supplementary Table S2.

### 4.11. Western Blot Analysis

The amount of protein in the cytoplasm and membrane was quantitated according to the Bradford method (Bradford 1976). The proteins were loaded onto a 10% SDS–polyacrylamide gel, and electrophoresis was carried out in a Tris-glycine buffer for 1 h. After transferring to nylon membranes and checking the position of the bands with a Ponceau solution, the membranes were blocked (5% skim milk in TBS, 0.1% Tween-20) at room temperature for 60 min and then incubated with the primary antibody overnight at 4 °C. Each primary antibody was diluted with 5% skim milk, as listed in Supplementary Materials Table S3. After washing, the membrane was incubated for 30 min in TBST buffer (25 mM Tris-base, 155 mM NaCl, and 0.1% Tween-20) and incubated with anti-rabbit lgG (Amersham, UK) or anti-goat lgG (ABcam, Cambridge, MA, USA) secondary antibody at room temperature for 1 h. The membrane was washed with TBST buffer for 30 min. Immunoreactive bands were developed by using an ECL kit (Pierce Chemical Co., Rockford, IL, USA), and the molecular weight of the bands was confirmed. A list of the antibodies used is provided in Supplementary Materials Table S3.

### 4.12. Statistical Analysis

Data are expressed as the mean ± standard error (SE). All statistical analyses were performed by using IBM SPSS Statistics 26 (SPSS, Inc., Chicago, IL, USA). Statistical differences between the YC and AC results were performed by the Mann–Whitney U test. A Kruskal–Wallis test was used to analyzed differences among the aging groups, followed by Dunn’s Multiple Comparison test. Data with different superscript letters are significantly different according to the Kruskal–Wallis post hoc test; *p*-values less than 0.05 were considered statistically significant.

## 5. Conclusions

In summary, we demonstrated that a 12-week supplementation with CP and CTP improved age-associated sarcopenia by modulating the molecular signals involved in protein synthesis in different ways. Specifically, CTP supplementation significantly increased the muscle thickness from the fourth week and expression of activated Akt and mTOR compared to the AC and CP group. CP supplementation increased the muscle thickness from the eighth week and increased the activated PI3K and AMPK expression. Thus, CP and CTP supplements may represent an effective therapeutic approach for age-associated sarcopenia by increasing IGF-1 and decreasing myostatin expression.

## Figures and Tables

**Table 1:** Effect of a 12-week supplementation of CP and CTP on body weight gain, food intake, food efficiency ratio, and organ weight.

	YC	AC	CP	CTP
	Mean ± SE	Mean ± SE	Mean ± SE	Mean ± SE
Initial BW (g)	24.70 ± 0.48 ***	36.50 ± 0.47	35.98 ± 0.64	36.27 ± 0.41
Final BW (g)	30.06 ± 0.50 ***	42.01 ± 0.85	40.47 ± 0.87	40.40 ± 0.87
Total BWG (g)	5.36 ± 0.24	5.52 ± 0.67	4.11 ± 0.63	3.78 ± 0.89
Food Intake (g/day)	3.17 ± 0.08 *	3.50 ± 0.09	3.34 ± 0.06	3.32 ± 0.08
Energy Intake (kcal/day)	12.53 ± 0.32 *	13.82 ± 0.34	13.19 ± 0.25	13.09 ± 0.30
FER	0.04 ± 0.00	0.03 ± 0.00	0.30 ± 0.00	0.03 ± 0.01
Liver (g/100 g BW)	3.16 ± 0.05 **	3.97 ± 0.09	3.23 ± 0.06 ^&&^	3.24 ± 0.06 ^$$^
Kidney (g/100 g BW)	1.00 ± 0.01 **	0.89 ± 0.01	0.97 ± 0.02 ^&&^	1.02 ± 0.04 ^$$^

Data are the mean ± SE. Significant differences between YC versus AC are indicated as follows: * *p* < 0.05, ** *p* < 0.01, and *** *p* < 0.001. Significant differences between CP versus AC are indicated: ^&&^ *p* < 0.01. Significant differences between CTP versus AC are indicated: ^$$^ *p* < 0.01. YC, young mice control (12 weeks old, *n* = 6); AC, aging control (60 weeks old, *n* = 6); CP, collagen peptide (60 weeks old, *n* = 6); CTP, collagen tripeptide (60 weeks old, *n* = 6); BW, body weight; BWG, body weight gain; FER, food efficiency ratio = body weight gain/energy intake per day.

## Data Availability

Data available upon request, due to restrictions, e.g., privacy or ethical.

## Supplementary Materials

The following supporting information can be downloaded at: https://www.mdpi.com/article/10.3390/molecules27092718/s1, Table S1. Diet composition for animal experiment; Table S2. Primer sequences for RT-qPCR; Table S3. Western blot antibody.

## Sample Availability

Samples of the compounds are available from the authors and Amicogen, Inc. (Jinju, Korea).
