# Supplementary material for: A Collagen Hydrolysate Containing Tripeptides Ameliorates Sarcopenia in Middle-Aged Mice
Source: Molecules. 2022 Apr 22;27(9):2718. doi: 10.3390/molecules27092718 (PMC9104253; doi:10.3390/molecules27092718)
Supplement: Supplementary file 1 [file molecules-27-02718-s001.zip › molecules-1689593-supplementary.pdf]

A Collagen Hydrolysate Containing Tripeptides Ameliorates Sarcopenia in Middle-Aged Mice

Ji-Eun Kim [1,2,3,†], Eun-Young Kwon [1,2,3,†] and Youngji Han [1,2,3,*]

Supplementary Materials Table S1. Diet composition for animal experiment.

Ingredient(g)	YC (AIN-93G)	AC (AIN-93G)	AC + 0.2% CP	AC + 0.2% CTP
**Casein**	200	200	200	200
**Corn Starch**	397.5	397.5	397.5	397.5
**Sucrose**	100	100	100	100
**Maltodextrin**	132	132	132	132
**Cellulose**	50	50	**48**	**48**
**Soybean Oil**	70	70	70	70
**Mineral Mix**	35	35	35	35
**Vitamin Mix**	10	10	10	10
**TBHQ, antioxidant**	0.014	0.014	0.014	0.014
**L-Cystine**	3	3	3	3
**Cholin Bitartrate**	2.5	2.5	2.5	2.5
**Collagen peptide**	-	-	**2**	-
**Collagen tripeptide**	-	-	-	**2**
**Total (g)**	1000	1000	1000	1000
**Calorie (cal/g)**	3948	3948	3956	3956

YC, Young mice control (6-week-old); AC, Aging control (48-week-old); CP, Collagen peptide (48-week-old); CTP, Collagen tripeptide (48-week-old)

*__Mineral Mixture (AIN-93G) (gram/kg)__: Calcium Carbonate 357, Monopotassium Phosphate 196%, Potassium Citrate Monohydrate 70.78, Sodium Chloride 74, Potassium Sulfate 46.6, Magnesium Oxide 24, Ferric Citrate 6.06, Zinc Carbonate 1.65, Manganese Carbonate 0.63, Copper Carbonate 0.3, Potassium Iodate 0.01%, Sodium Selenate, Anhydrous 0.0103, Ammonium Molybdate.4H2O 0.00795, Sodium Metasilicate 9H2O 1.45, Chromium Potassium Sulfate.12H2O 0.275, Lithium Chloride 0.0174, Boric Acid 0.08145, Sodium Fluoride 0.0635, Nickel Carbonate 0.0318, Ammonium Vanadate 0.0066, Powdered Sugar 221

†**Vitamin mix (AIN-93G) (gram/kg)**: Nicotinic Acid 3.00, D-Calcium Pantothenate 1.60, Pyridoxine HCl 0.70, Thiamine HCl 0.60, Riboflavin 0.60, Folic Acid 0.20, D-Biotin 0.02, Vitamin B12 (0.1% triturated in mannitol) 2.50, a-Tocopherol Powder (250 U/gm) 30.00, Vitamin A Palmitate (250,000 U/gm) 1.60, Vitamin D3 (400,000 U/gm) 0.25, Phylloquinone 0.075, Powdered Sucrose 959.655

Supplementary Materials Table S2. Primer sequences for RT-qPCR.

**Gene (Gene ID)**	**Sequence**
GAPDH**(14433)**	F- TGC AGT GGC AAA GTG GAG AT
	R- TTG AAT TTG CCG TGA GTG GA
Atrogin-1**(67731)**	F- AAC CGG GAG GCC AGC TAA AGA ACA
	R- TGG GCC TAC AGA ACA GAC AGT GC
FoxO3**(56484)**	F- TCG CCT CCT GGC GGG CTT A
	R- ACG GCG GTG CTA GCC TGA GA
Mef2**(17260)**	F- ACA CGC ATA ATG GAT GAG AGG AAC CGA C
	R- CAA CGA TAT CCG AGT TCG TCC TGC TTT C
Myf5**(17877)**	F- AGG AAA AGA AGC CCT GAA GC
	R- GCA AAA AGA ACA GGC AGA GG
Myf6**(17878)**	F- CAA GAA AAT CTT GAG GGT GCG G
	R- TTA GCC GTT ATC ACG AGC CC
MyoD**(17927)**	F- GCT TCT ATC GCC GCC ACT CC
	R- CGC ACA TGC TCA TCC TCA CG
Myogenin**(17928)**	F- CCT TGC TCA GCT CCC TCA
	R- TGG GAG TTG CAT TCA CTG G
MuRF1**(4433766)**	F- GAG AAC CTG GAG AAG CAG CT
	R- CCG CGG TTG GTC CAG TAG

GAPDH, Glyceraldehyde-3-phosphate dehydrogenase; *FoxO3*, Forkhead box O3; *Mef2*, Myocyte enhancer factor-2; *Myf5*, Myogenic factor 5; *Myf6*, Myogenic factor 6; *MyoD*, Myogenic differentiation factor; *MuRF1*, Muscle ring-finger protein-1

Supplementary Materials Table S3. Western blot antibody.

Primary antibody	Primary antibody dilution	Secondary antibody	Secondary antibody dilution
**GAPDH**	1:1000	anti-rabbit lgG	1:10000
**PGC1α**	1:1000	anti-rabbit lgG	1:10000
**p-Akt**	1:1000	anti-rabbit lgG	1:10000
**Akt**	1:1000	anti-rabbit lgG	1:10000
**p-PI3K**	1:1000	anti-rabbit lgG	1:10000
**PI3K**	1:1000	anti-rabbit lgG	1:10000
**p-AMPK**	1:1000	anti-rabbit lgG	1:10000
**AMPK**	1:1000	anti-rabbit lgG	1:10000
**mTOR**	1:1000	anti-rabbit lgG	1:10000
**TNF-α**	1:1000	anti-rabbit lgG	1:10000

GAPDH, Glyceraldehyde-3-phosphate dehydrogenase; PGC1α, Peroxisome proliferator-activated receptor gamma coactivator 1-alpha; p-Akt, Phospho-Protein kinase B; Akt, Protein kinase B; p-PI3K, Phospho-phosphoinositide 3-kinase; PI3K, Phosphoinositide 3-kinase; p-AMPK, Phospho-AMP-activated protein kinase; AMPK, AMP-activated protein kinase; mTOR, mechanistic target of rapamycin; TNF-α, Tumor necrosis factor-alpha.
